# Enhancing Bone Scaffold Fabrication: A Comparative Study of Manual Casting and Automated 3D Bioprinting

**DOI:** 10.1007/s10439-025-03752-9

**Published:** 2025-06-05

**Authors:** Yasser Ahmed, Ali S. Alshami, Ashraf Al-Goraee, Collins P. Obeng, Rebecca Kennedy, Hesham Abdelaziz, Ryan Striker

**Affiliations:** 1 Biomedical Engineering Department, University of North Dakota, Grand Forks, ND 58202 USA; 2 Department of Chemical Engineering, University of North Dakota, Grand Forks, ND 58202 USA

**Keywords:** Bioprinting, 3D printing, Automation, Bone regeneration, Tissue engineering, In vitro

## Abstract

While fabrication of bone scaffolds is important for the development of tissue engineering, traditional techniques have typically been prone to either scaling or reproducibility issues. This paper highlights a strategy for automated 3D printing and bioprinting techniques that enhance precision and efficiency in the production of PLGA–HA scaffolds. We realized significant improvements in efficiency, reproducibility, and scalability through optimization of 3D printing parameters, improvement of material handling, and refinement of the fabrication process. Precise measurement consequently minimized material waste; the introduction of a mesh filter allowed for high-throughput experimentation without compromising the integrity of individual scaffolds, streamlining the workflow. Combining automated casting with state-of-the-art 3D bioprinting, our experimental methodology precisely applied the bioactive materials, reducing the processing time fivefold and enhancing precision. Besides, automated casting produced thicker, better-quality scaffolds averaging 0.02354 g, which is against 0.01169 g using the manual approach, effectively doubling the retention of the PLGA–HA coating on a PVA mold. Excellent cell viability and adhesion on automated scaffolds have been further underlined for application in tissue engineering during in vitro studies using multipotent mesenchymal stromal cells. Although conventional techniques, such as injection molding, are standard for large lots, 3D printing has advantages in scaffold fabrication regarding control over geometry and homogeneous material properties. Equally important, these characteristics are necessary to achieve repeatable and up-scaled experimental results.

## Introduction

Bone injuries are a global health concern. Approximately 178 million new bone fractures occur annually [1], and over 300,000 hip fracture cases occur in the United States annually [2]. One in four patients who experience severe hip fractures die within 1 year of treatment [3]. Bone fracture defects are not necessarily repaired with surgery alone; 521 of 16,872 patients required additional revision surgery for spinal complications from 2006 to 2018 [4].

Current research focuses on optimizing biomaterial properties and internal architecture to enhance bone regeneration [5–7]. The importance of the coating process for bone scaffold fabrication is significantly understated. While effective for initial testing, manual coating methods are time-consuming and prone to inconsistencies [8, 9], such as variable material viscosity, coverage, porosity, pore size, and morphological structures [10, 11]. To address these limitations, this study focuses on implementing an automated 3D printing method that enhances reproducibility and scalability in scaffold fabrication.

Bone scaffold fabrication is essential for providing a framework for cell attachment, proliferation, and differentiation; however, this technology is limited by conventional fabrication methods, where the inefficiencies constrain scalability and reproducibility. An innovative approach is needed to streamline the process, enhance precision, and facilitate large-scale experimentation [12].

Current bone scaffold fabrication uses manual coating to incorporate bioactive materials or insert cells onto the bone scaffold molds [13], which causes variations in material viscosity, thickness, and uniformity, especially in complex geometries [14, 15]. Manual coating is a labor-intensive process that dramatically impacts the ability to scale and streamline scaffold production [14, 16].

Research has revealed that coating material viscosity and the coating technique employed by the operator can affect the uniformity of the protein films on biomaterial surfaces, which can have a negative effect on cell attachment and proliferation [17, 18]. One avenue that has been explored is combining additive manufacturing and electrospinning for scaffold fabrication [19, 20]. These studies examined the limitations associated with manual coating and the need for bioactive material deposition control [19, 20]. Other studies have investigated the importance of controlling bioactive molecule release by implementing efficient and accurate coating methods [21, 22]. 3D printing has been used to further improve the precision and complexity of designs that are fabricated with controlled surface properties to enhance cell interactions [23]. Bioprinting advancements address the limitations of manual coating by allowing precise material deposition control, which allows for consistent coating thickness and scaffold performance uniformity [24, 25].

Substantial research was conducted that focuses on developing bone scaffold fabrication techniques. An example is a study that investigated bioactive composite scaffolds based on collagen, gelatin, and nano-β-TCP for alveolar bone regeneration [26]. The impact of nanostructure was studied in fabrication of highly ordered willemite/PCL bone scaffolds using 3D printing on mechanical properties in vitro behavior has been examined in another study [27]. Gelatin/monetite electrospun scaffolds for bone tissue regeneration have also been explored, focusing on their fabrication, characterization, and in vitro evaluation [28]. There were also 3D permeable scaffolds from CaP/PEGDA hydrogel biocomposites that were developed for bone grafting using stereolithography, highlighting their potential for clinical application [29].

This work, therefore, presents an experimental design that focuses on how the PLGA–HA scaffold fabrication could be further improved by introducing automation and precision control into a specific process of solvent evaporation. For the purposes of the present discussion, we will call this automatic layer-by-layer fabrication technique the building scaffold structure using ‘3D printing.’ A subcategory of 3D printing, ‘bioprinting’ is a method by which bioactive material is deposited to allow cellular attachment and proliferation. Bioprinting is applied in our automated casting method to fabricate scaffolds in a more accurate and bioactive way. Previous methods form the basis on which new automation techniques are applied in order to solve the specific problems related to consistency and reproducibility of scaffolds [30]. Other studies have discussed improvements in experimental procedures in various respects, from material handling down to process optimization, 3D printing, and automated casting. The problems of chloroform volatility are avoided by the material handling techniques we describe: we successfully avoided the problems inherent in this issue by moving onto weight-based measurements instead of volume-based and by using chemically inert materials, like borosilicate glass and polypropylene. To alleviate the scalability problem, we designed a mesh filter that could enable the experimentation of multiple scaffolds, while maintaining individual identity. Our method of automated casting using bioprinting technology is considered a major breakthrough compared to conventional methods. This is because automation combines with innovation in enhancing efficiency, consistency, and scalability [30]. In this paper, we will call scaffolds made by the automated casting method automated scaffolds and those made by manual casting manual scaffolds.

## Materials and Methods

### Experimental Procedure

We began examining optimized scaffold fabrication by seeking to control the challenge of material handling. The primary challenge in material handling was chloroform volatility [30]; therefore, we used borosilicate glass vials with polypropylene lids to mitigate the risk of chemical reactions and enhance experiment accuracy [31]. Chloroform volatility required replacing volume-based measurements with weight-based measurements.

A mesh filter for the well plate tops was developed and 3D printed to address the scalability issue. This technique allowed us to study multiple scaffolds, while maintaining individual identity in measurements along various stages of experimentation, increasing experimental bandwidth and enhancing workflow efficiency [32].

Our approach’s greatest contribution is integrating automated casting using state-of-the-art 3D printing technology, with bioprinting used to deposit bioactive material for scaffolds [33]. A Lulzbot bioprinter was used to achieve elevated levels of precision and consistency in scaffold fabrication. To verify the proposed method, a comparison was conducted between manual and automated casting in terms of resultant weight retention, casting time, morphology, and structural integrity. Fourier transform infrared spectroscopy (FTIR) confirmed no alterations in chemical composition, and scanning electron microscopy (SEM) verified consistent pore structures. Meticulous design iteration and parameter optimization allowed us to successfully automate complex casting procedures, reducing the reliance on skilled personnel and minimizing processing time.

### Scaffold Fabrication

**Fig. 1:**
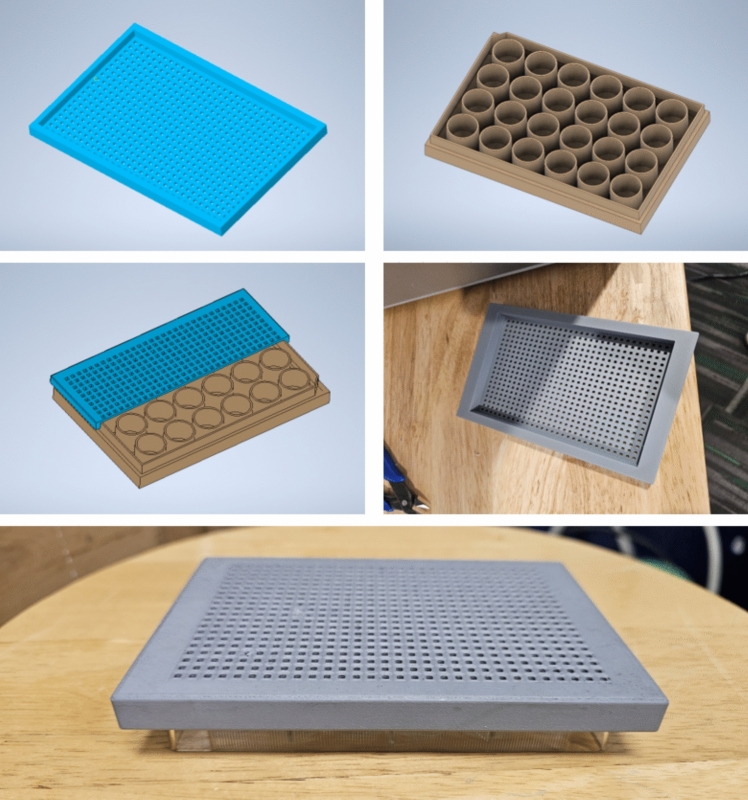
Design and fabrication of the PLA mesh filter for a 24-sample well plate

A porous 3D scaffold was fabricated using PLGA and hydroxyapatite (HA) nanoparticles through casting and solvent evaporation. The experimental procedure enhancements include the following:

1. *Chloroform handling* borosilicate glass vials with polypropylene lids were utilized for storage and handling to mitigate reactivity and evaporation issues.
2. *Precise chloroform measurement* a weight-based approach was adopted because the chloroform rapidly evaporated during handling and measurement. This approach ensured that the required volume was attained even after accounting for evaporation while measurements took place.
3. *Mesh design for scalability* a mesh filter was designed using Autodesk Inventor software and then 3D printed using PLA with the Bambu Carbon X1 3D printer to accommodate simultaneous experimentation with multiple molds. This process streamlined workflow and improved experimental throughput (Fig. 1).
4. *Automated casting process with a 3D bioprinter* the most significant advancement in the scaffold fabrication process was integrating the Lulzbot bioprinter to automate the PLGA–HA solution casting. The casting path was constructed using Autodesk Inventor software. Iterative refinements were performed to optimize functionality and volume. The CURA Lulzbot Edition slicer software was utilized to fine-tune extruder parameters, ensuring precise and consistent casting. The ideal settings for our design included an extrusion rate of 4 mm/s and a layer height of 2 mm, determined after experimentation and slicer parameter optimization. The Lulzbot facilitated casting process automation, eliminating the need for personnel trained in casting and significantly reducing time. This design parameter optimization enabled us to coat four molds using 0.5 mL (about 0.02 oz) of the PLGA–HA solution in 5 min and 11 s using the bioprinter’s 2.5 mL (approximately 0.08 oz) syringe. Four molds were cast in 3 min and 46 s using the 5 mL (about 0.17 oz) syringe. These times prove the system’s efficiency compared to the 10 min and 51 s needed to cast only two molds manually.

### Experimental Setup

Scaffold fabrication began with preparing polyvinyl alcohol (PVA) molds. The PVA was stored under controlled humidity conditions. A G-code file was fed to an Ender 3D printer to print the mold. The initial weight of the individual molds was measured before storage in their respective slots in the well plate.

Solution synthesis began with placing an empty borosilicate glass vial inside a lab precision scale for calibration. Chloroform was carefully added to the vial until the scale read 5.92 g. Poly(dl-lactide-*co*-glycolide) (PLGA) was stored in a freezer (−15 to − 18 °C) and then weighed on the precision scale to extract 100 mg, which was then added to the vial. A magnetic stirring bar was inserted into the solution. The round bottom flask was corked and sealed with parafilm before it was placed on top of a hotplate stirrer without adding heat for at least 3 h until the PLGA dissolved completely. We then added hydroxyapatite (nHA) slowly, while dispersing it using a probe sonicator for 2 to 3 min to ensure even nanoparticle distribution.

**Fig. 2:**
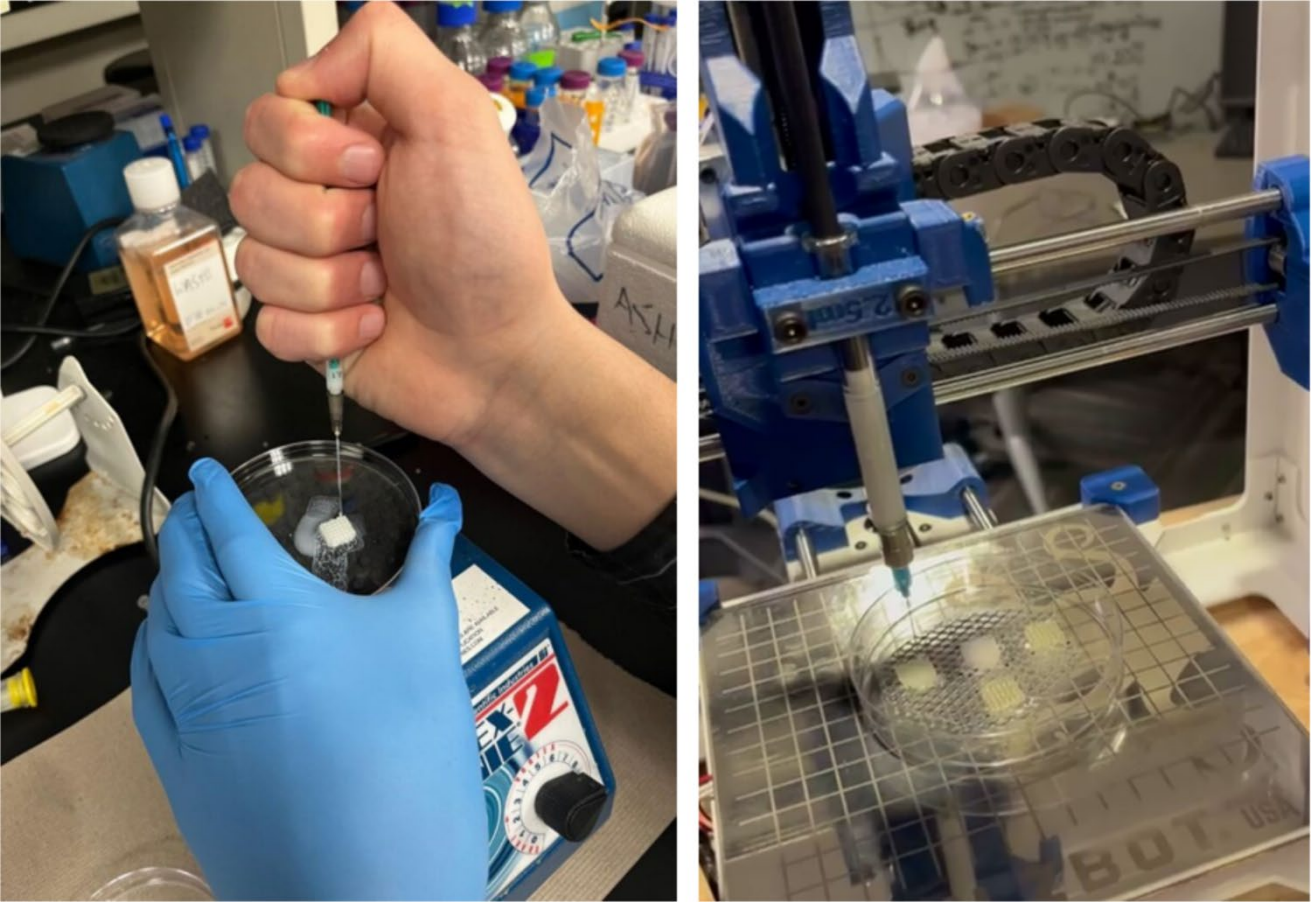
Manual vs. automated PLGA–HA solution casting on PVA molds. Manual casts were extruded sequentially on a vortex mixer for distribution. Automated casting occurred in batches without a mixer

Casting involved designing the bioprinter’s 3D path using 3D CAD software. The Lulzbot bioprinter was used to apply the PLGA–nHA solution on the PVA molds. The bioprinter syringe was loaded with the solution and placed in the bioprinter’s syringe housing. The printer was then calibrated. A metallic mesh elevated the molds from the bioprinter floor to ensure total coverage. The solution was then cast on the molds (Fig. 2). The bioprinter can cast up to eight molds in one run. The molds were left to dry after casting to let the chloroform evaporate completely, leaving the remaining PLGA–HA coating. Each mold was weighed post-drying, and the difference between the resultant and original mold weights was noted.

De-ionized water was added to the wells, and a PLA filter was placed atop the well plate. The well plate was placed in an incubator (shaker) at 100 rpm and kept at room temperature. Water was periodically replaced until the PVA dissolved completely. The molds were then left to dry and weighed to verify dissolution.

### In Vitro Experimentation

We sourced human chorionic membrane mesenchymal stromal cells (HCMSC) from ScienCell Research Laboratories for the in vitro experiment. The cell culture procedure was followed according to the supplier’s standard protocol. Cells were cryopreserved at the first passage and stored in liquid nitrogen prior to use. 10 mL of sterile Dulbecco’s phosphate buffered saline and 150 μL of fibronectin stock solution (ScienCell Cat. #8248) were inserted into T-75 flasks before the cells were removed for storage. The flask was then incubated overnight at 37 °C. The cells were placed in a water bath at 37 °C and then into the prepared flask with 20 mL of mesenchymal stem cell medium (MSCM, ScienCell Cat. #7501) to prepare for culture. The cells were then incubated at 37 °C with 5% CO_2_ in the PHCBI MCO-170AICUVL incubator.

Cells were observed daily under the Olympus CKX41 microscope. The cells were passaged at approximately 90% confluency. The procedure for passaging is as follows: aspirating the medium, inserting 5 mL of DPBS into the flask, accompanied by 5 mL of trypsin/EDTA solution, then placing the cells into the incubator for 5 min for suspension. The suspended cells were collected with the DPBS and trypsin/EDTA in a 50 mL conical tube containing 5 mL of fetal bovine serum (FBS, ScienCell Cat. #0500). The remaining cells were extracted by adding 5 mL of trypsin neutralization solution (TNS, ScienCell Cat. #0113) into the flask and incubated for three more minutes. The flask was emptied once more into the conical tube.

The cells were separated from the solution in the conical tube after cell collection was completed. The tube was placed in an Eppendorf 5702 centrifuge and left for 5 min at 1000 rpm for separation. The supernatant was then aspirated, and the cells were suspended within the tube in 10 mL of culture media (MSCM). A DeNovix cell counter was utilized to count cells using 5 μL of cell suspension mixed with 5 μL of trypan blue per run. A total of two runs were performed.

Twelve bone scaffolds (6 Automated and 6 Manual) were prepared on a well plate for cell seeding. The scaffolds were sterilized with a 70% ethanol solution. 0.5 mL of cell suspension medium was inserted in each well, and the plate was incubated at 37 °C with 5% CO_2_.

### Characterization

#### Casting Time Analysis

An essential aspect of the automation procedure was determining the scale of the bone scaffold production casting time. The time it took to load the PLGA–HA solution into the syringe or bioprinter extruder syringe, cast the material on the PVA molds evenly, and then replace the scaffolds was measured by recording the entire duration with a stopwatch. The time needed for the manual casting process included recording the time it took three trained individuals to make six casts each, with the average time used to account for variance. There were five replicate casts during automated casting.

#### Scaffold Weight Measurement

The second parameter measured in the experiment was the difference in material retained during manual vs. automated casting. A Denver Instrument M-120 high-precision scale was employed to weigh the molds and assign them to their respective wells in the 24-well plate. The weights of the PVA molds pre-casting, the molds with the PLGA–HA cast on them, and the weights of the scaffolds post-PVA dissolution were measured individually. Twelve scaffolds were manually cast on each well plate, and 12 were automatically cast for accurate comparison. The goal was to establish the difference in manual vs. automated weight retention and to assure the automated procedure’s repeatability and reproducibility.

#### Fourier Transform Infrared Spectroscopy (FTIR)

FTIR spectra were obtained to identify the material’s functional groups and analyze the chemical bonds of the PLGA–HA [34]. The FTIR also guaranteed that the results from the automated casting method matched those of manual casting, ensuring that no contaminants were introduced in the enhanced procedure and that the existing functional groups were not compromised. The Thermo-Scientific Nicolet iS5 spectrometer equipped with the OMNIC 8.2 software was used to measure the transmittance of the material. FTIR analysis was conducted to confirm that the automated casting method did not introduce any chemical changes or contamination. By comparing the spectra of manually and automatically cast scaffolds, we ensure that the process retains the integrity of the original PLGA–HA material.

#### Microstructure and Morphology

The microstructure, pore size, and morphology of the manual and automated PLGA–HA scaffolds were obtained with scanning electron microscopy, SEM [35] using the HITACHI S-4700 device equipped with its designated software. All samples were left to dry overnight before being coated with a ~ 5-nm-thin carbon layer and mounted on aluminum stubs in preparation for scanning. Various magnification values were used to measure surface properties on a macro and micro-scale. The goal was to characterize the properties and confirm the similarity in results for both fabrication methods. Morphological analyses revealed the scaffolds’ geometry and macroscopic structures. Pore size affects osteointegration, cellular behaviors, and bone regeneration [36]. Porosity facilitates nutrient distribution and cellular infiltration within the cellular matrix [37]. Microstructure observations provided insight into essential details, such as interconnectivity and surface topography in cell proliferation and adhesion [38].

#### Mechanical Properties

A bone scaffold must have a hierarchal porous structure to mimic the complex extracellular matrix [39]. Sufficient mechanical strength is needed to mimic the bone’s environment for osteogenesis to occur, allowing the scaffold to maintain the required strength, while bone regeneration occurs. A compression test was needed to determine the elastic modulus and test the bone scaffolds’ mechanical properties. The compression test was conducted on the Shimadzu AGS-X Universal Testing Machine, equipped with a 5 kN loading cell and using the TrapeziumX 1.5.1 software. Compression was applied at a speed of 1 mm/min, and the maximum compression force was set to 300 N for safety. The test was standardized for consistency. The compression modulus was calculated from the linear region of the curves.

#### Cell Viability and Morphology

Cell viability and morphology were assessed using an Evos Floid microscope. The microscope was used to examine the bone scaffolds and cells immediately after seeding, after 24 h, and after 72 h. Morphological changes exhibited by the cells were observed and captured over the 72-h period. The expected results were cell spreading and flattening to indicate adherence to the bone scaffolds, adopting the spindle-like shape of mesenchymal stromal cells.

## Results

### Scaffold Fabrication Comparison

**Fig. 3:**
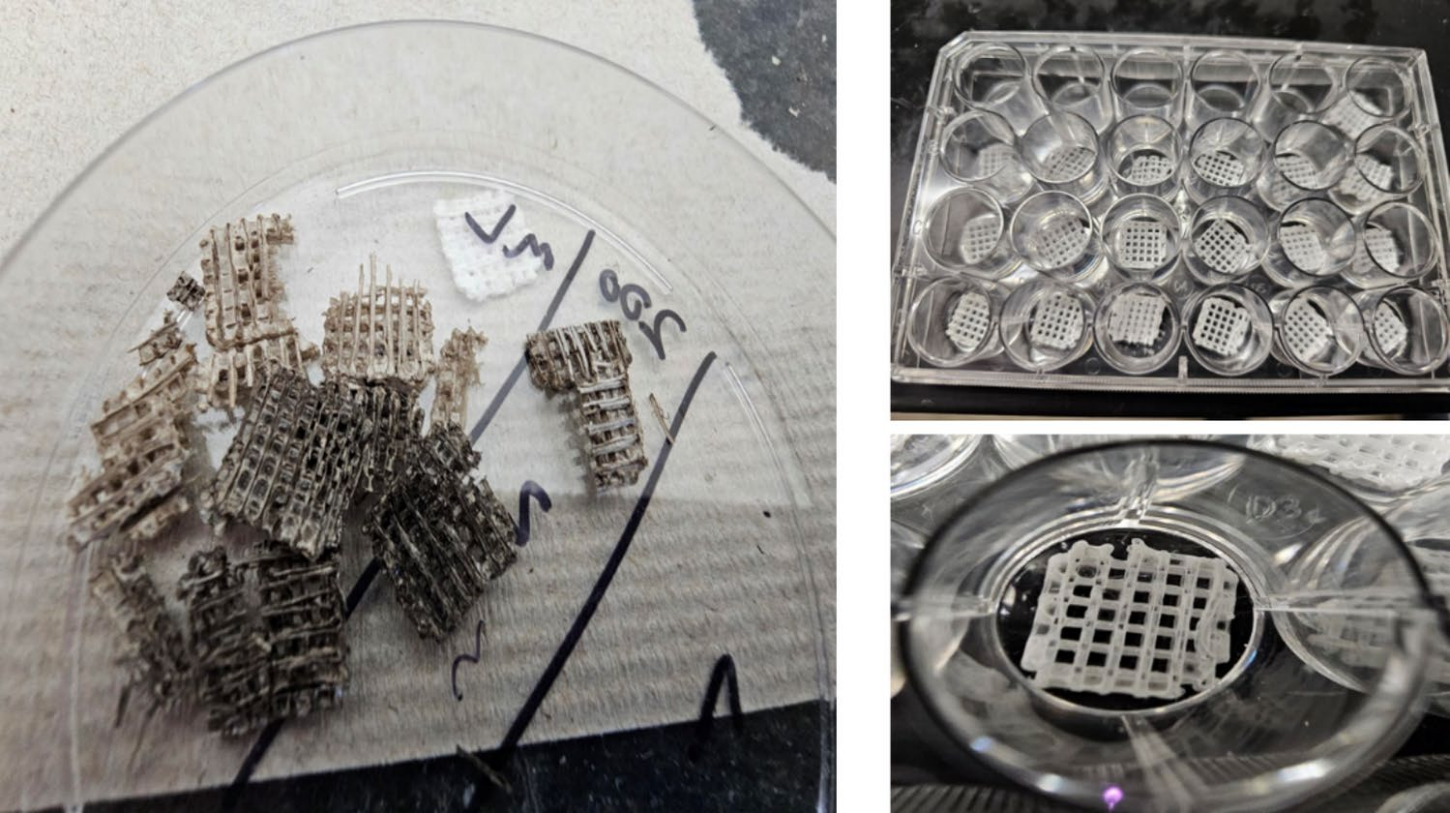
Comparison of scaffolds produced through manual casting (left, showing soggy, and inconsistent structures) versus automated casting (right, uniform, and reproducible scaffolds), demonstrating the structural consistency achieved with automation

The automated casting procedure using the Lulzbot bioprinter yielded significant improvements in scaffold fabrication compared to manual casting methods. The qualitative analysis revealed that automated casting produced more consistent scaffold weights and textures, while manually cast molds varied in appearance, with some molds appearing soggy and inconsistent in weight or color. Figure 3 shows defective samples from the manually cast scaffolds versus the consistent samples from the automated casts. The quantitative comparison of scaffold weights, which further supports the advantages of automation, is presented separately in Sect. 5.3.

The integration of a PLA filter in the dissolution process streamlined operations and facilitated the efficient removal of polyvinyl alcohol (PVA) mold remnants. Automated scaffold fabrication significantly improved efficiency and reproducibility, with greater control over scaffold geometry and material distribution.

### Casting Time Analysis

**Fig. 4:**
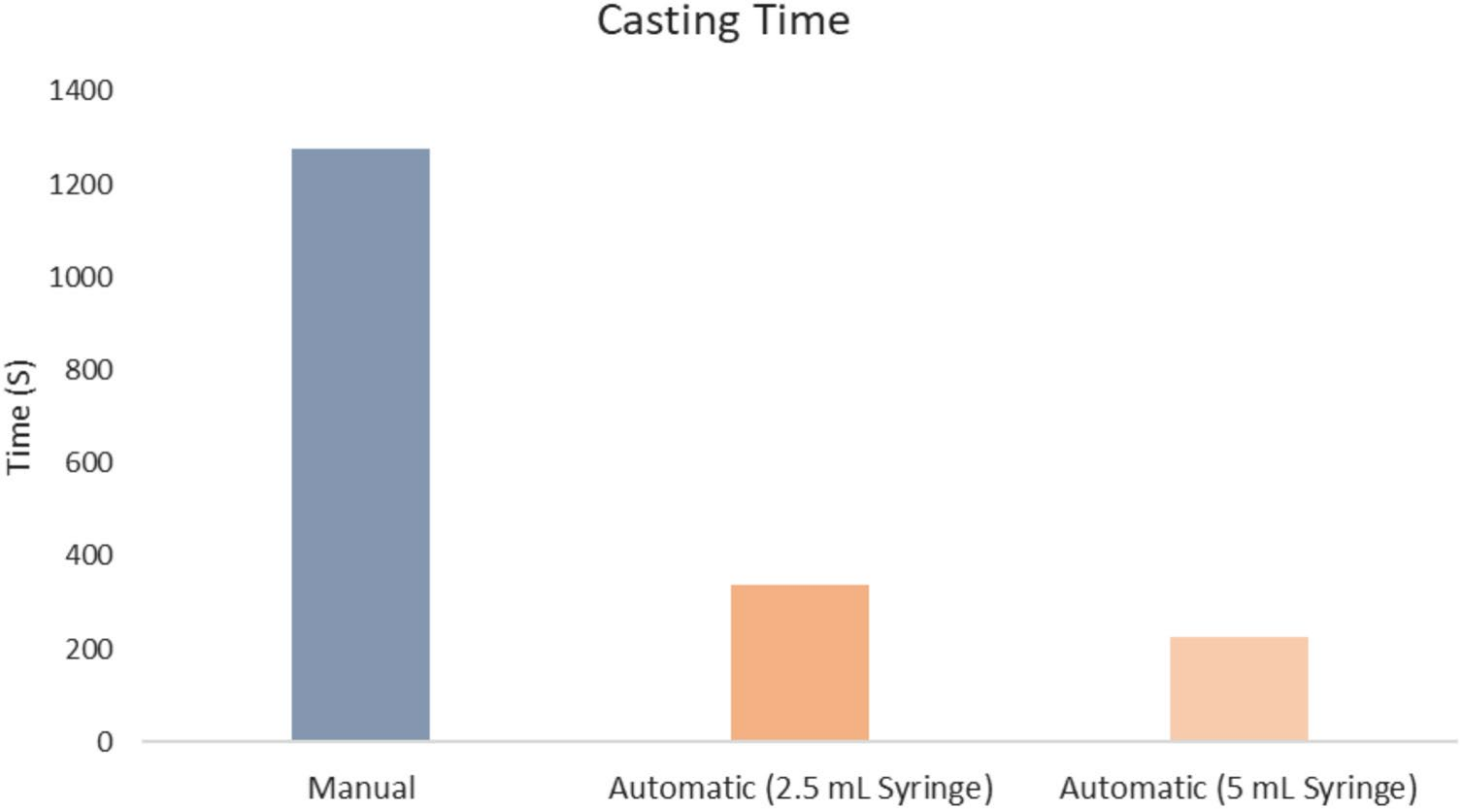
Time taken to cast four scaffolds using the manual method, automated with a 2.5 mL syringe, and automated with a 5 mL syringe

Figure 4 illustrates the average casting times based on five trials per method: manual casting required 10 min 51 s (655.8 ± 20.3 s), automated casting with a 2.5 mL syringe took 5 min 11 s (307.8 ± 14.9 s), and automated casting with a 5 mL syringe took 3 min 46 s (225.2 ± 6.6 s). We performed a one-way Analysis of Variance (ANOVA) to determine if there were any statistically significant differences among the three groups, followed by Tukey’s Honestly Significant Difference (HSD) post hoc test for pairwise comparisons. Both automated methods were significantly faster than manual casting (*p* < 0.05 for the 2.5 mL syringe, *p* < 0.01 for the 5 mL syringe). With that, the direct comparison between the two automated syringe sizes yielded a *p* value < 0.01, which meets the statistical significance threshold of *p* < 0.05. The Lulzbot bioprinter applies a constant extrusion force to the syringe plunger, meaning that the larger cross-sectional area of the 5 mL syringe allows it to deposit more material per unit time. Also the larger syringe can coat more scaffold molds in a single cycle, this contributes to the observed increase in efficiency.

### Scaffold Weight Measurement

**Fig. 5:**
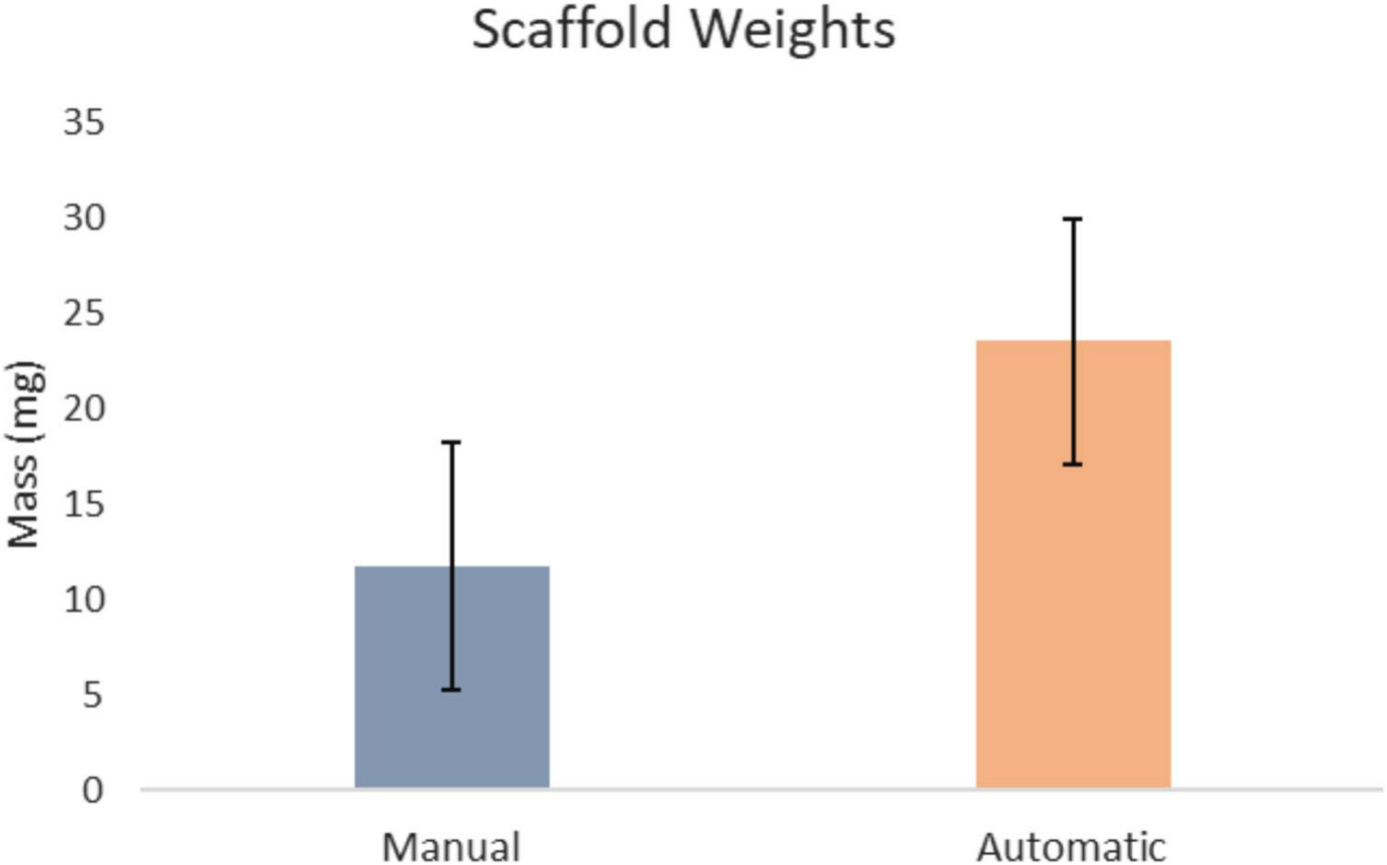
Average scaffold weights for manual casting (left) versus automated casting (right), showing greater material retention with automation

Scaffold weights from automated casting averaged 0.02354 g, while manual casting weights averaged 0.01169 g, a statistically significant difference (*p* < 0.01), indicating greater retention of the PLGA–HA coating with automation. A total of 12 manually cast scaffolds and 12 automated scaffolds were analyzed to ensure statistical reliability. Figure 5 compares the average scaffold weights of manual versus automated casting.

### Fourier Transform Infrared Spectroscopy (FTIR)

**Fig. 6:**
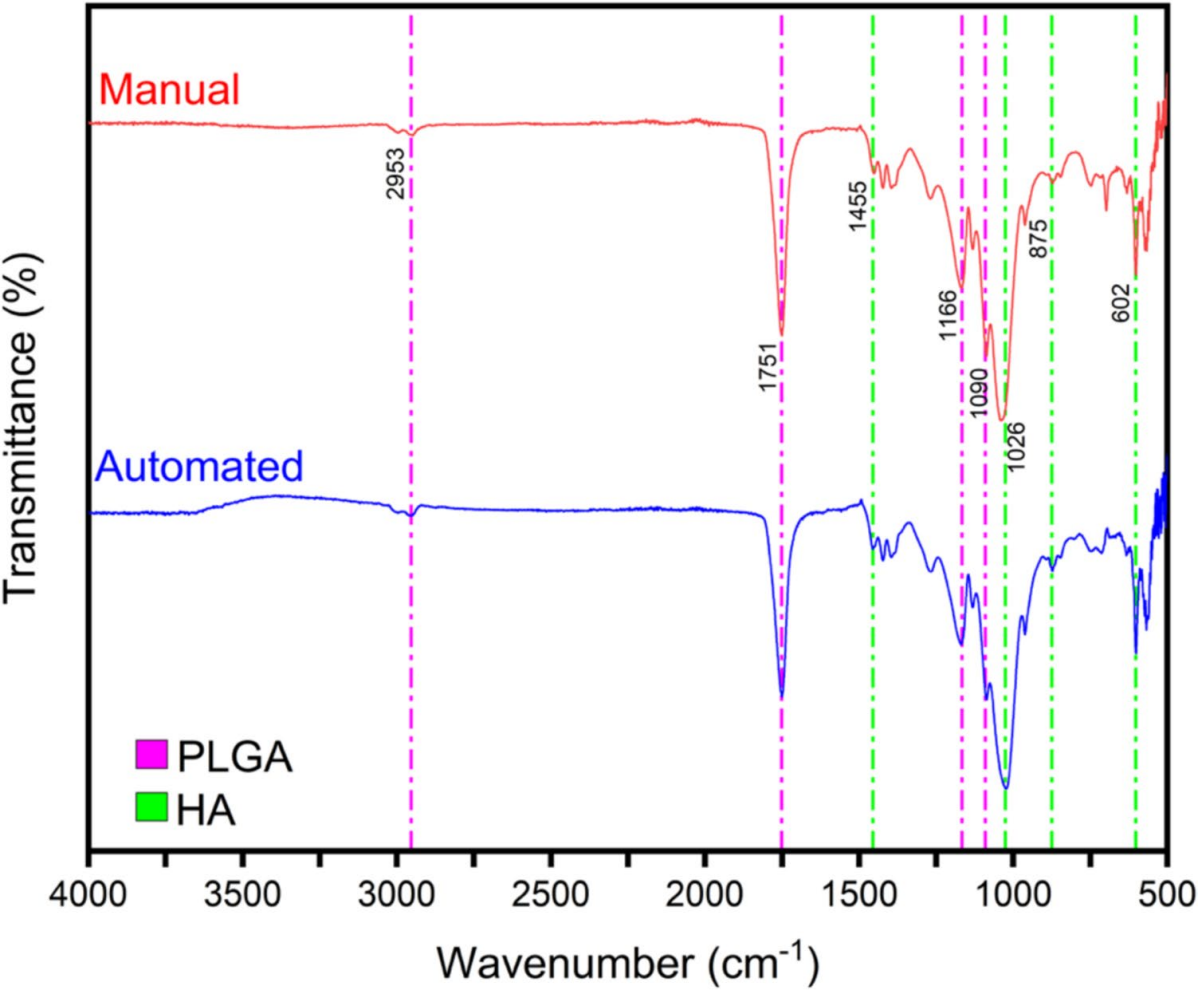
FTIR spectra of the manual and automated PLGA–HA scaffolds and functional group components

FTIR analyses were performed on both automated and manually cast scaffolds to confirm the chemical composition of the PLGA–HA. The FTIR spectra showed characteristic peaks for PLGA and HA, including peaks at 2953 cm^−1^, indicating C–H bonds in the methyl groups of PLGA, and a peak at 1751 cm^−1^, representing the carbonyl stretching of PLGA’s ester groups. Peaks at 1455 cm^−1^ represent O–H bond bending vibrations in HA, while phosphate vibrations appeared at 1026 cm^−1^ and 875 cm^−1^ [41, 42], confirming the integrity of the material in automated casting as shown in Fig. 6.

### Microstructure and Morphology

**Fig. 7:**
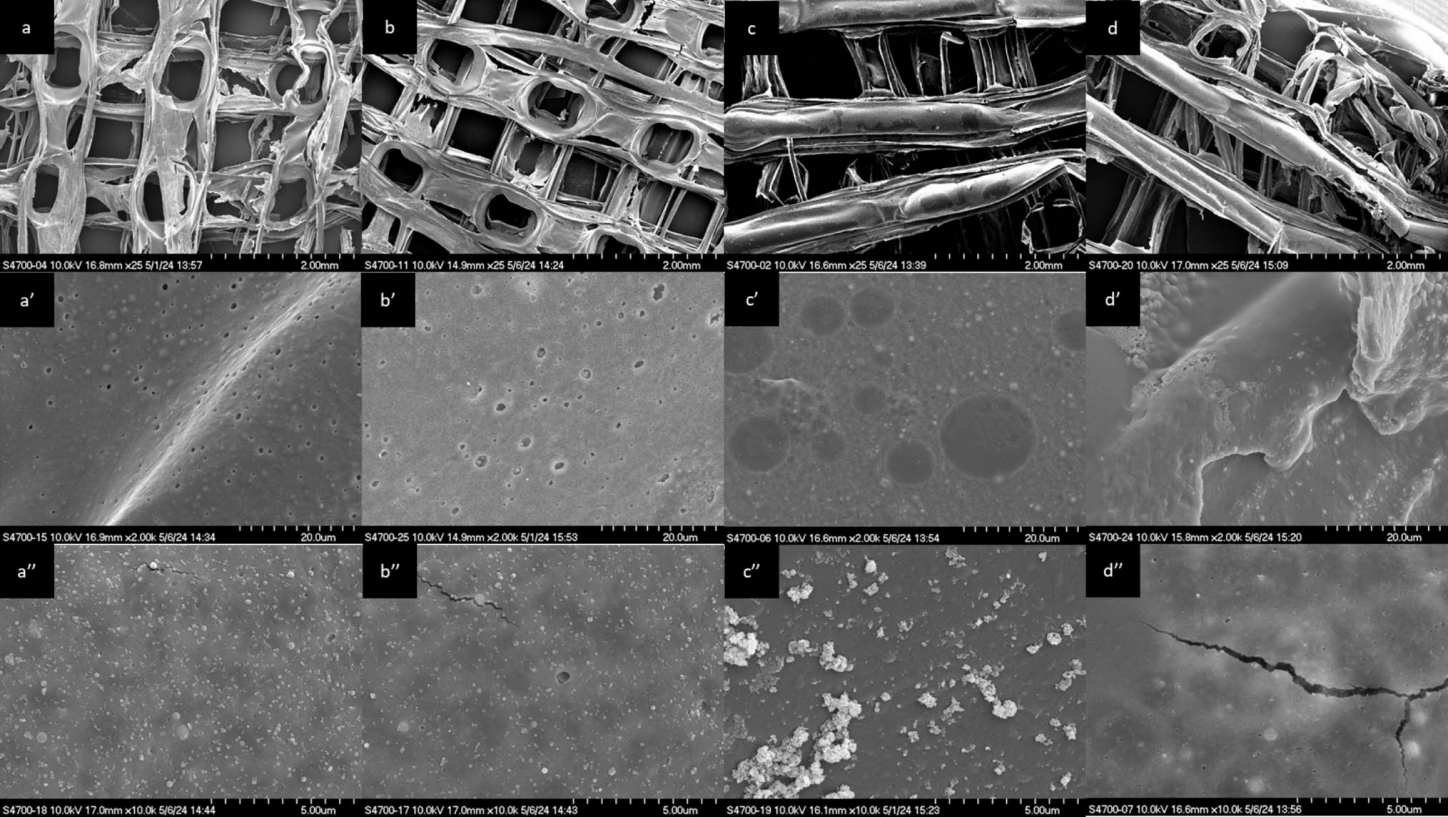
SEM images showing the microstructural differences between PLGA–HA scaffolds fabricated via automated casting (**a**, **a′**, **a″**; **b**, **b′**, **b″**) and manual casting (**c**, **c′**, **c″**; **d**, **d′**, **d″**) at magnifications of × 25, × 2000, and × 10,000, respectively

SEM imaging, presented in Fig. 7, captured the microstructure of PLGA–HA scaffolds fabricated by both methods at magnifications of × 25, × 2000, and × 10,000. The automated scaffolds exhibited consistency in pore sizes, ranging from 390 to 710 µm. In contrast, manual scaffolds showed variations in pore size from 251 to 1088 µm, along with irregular strands and a non-uniform structure. At × 2000 magnification, automated scaffolds exhibited a homogeneous topography with good HA particle dispersion. At × 10,000 magnification, automated scaffolds displayed a uniform texture, whereas manual scaffolds showed localized HA accumulations and cracks.

### Mechanical Properties

**Fig. 8:**
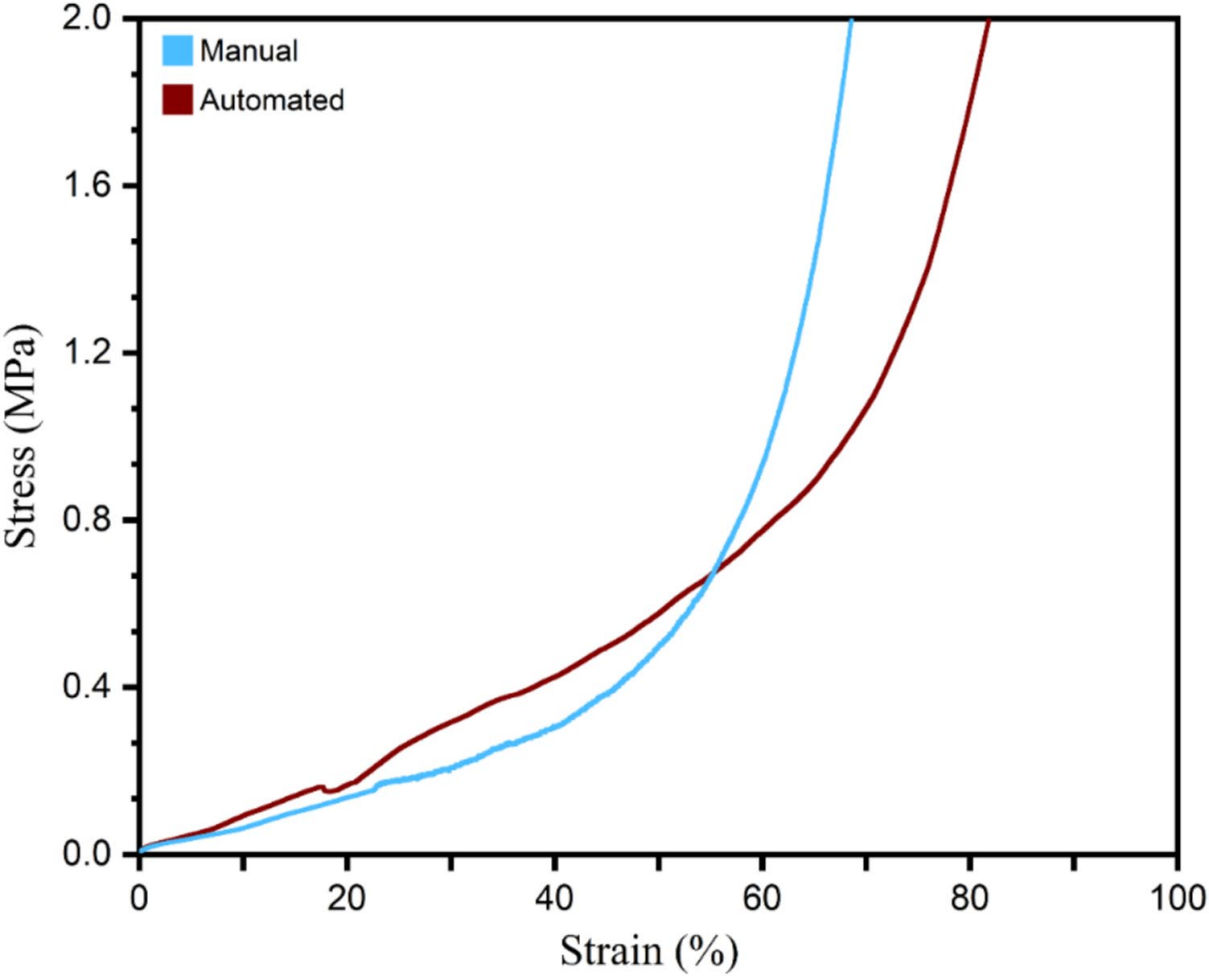
Mechanical compression curves for the manual and automated fabrication of the PLGA–HA scaffolds

Compression tests are widely used to measure bone scaffold stiffness [14]. Compression tests for both the manual and automated casts indicated maximum stress before failure of 1.6 ± 0.08 MPa. The elastic modulus observed in the automated scaffolds was approximately 0.77 MPa for automated scaffolds compared to 0.48 MPa for the manual scaffolds, with statistical significance (*p* < 0.05), indicating supporting the increased structural integrity of the automated scaffolds as shown in Fig. 8.

### Cell Viability and Morphology

**Fig. 9:**
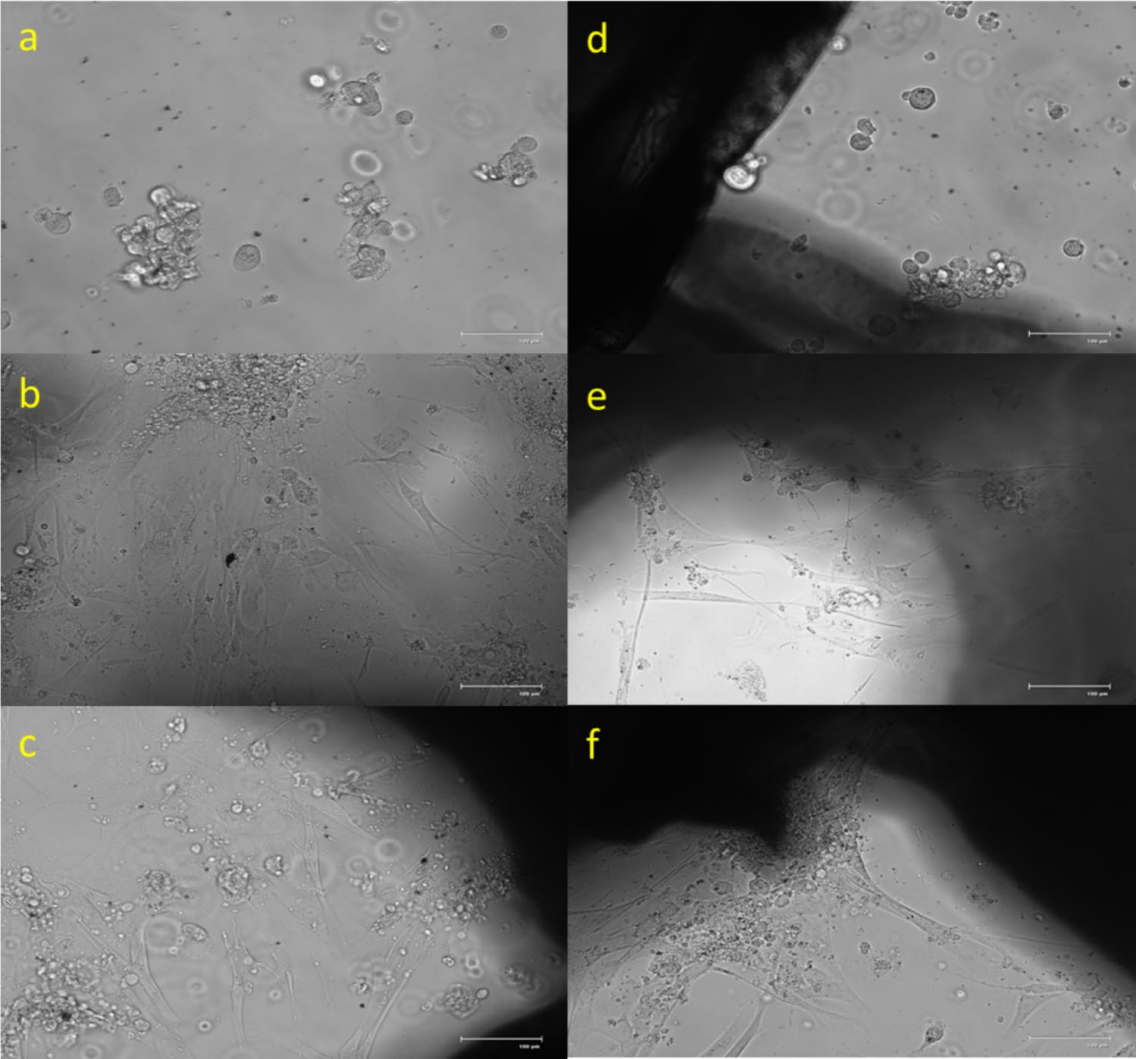
Microscopic images of cell-seeded bone scaffolds. **a**–**c** Represent images of the automated scaffolds at 0, 24, and 72 h, respectively. **d**–**f** Represent images of the manual scaffolds at 0, 24, and 72 h, respectively

Cell counts averaged 326,000 live cells/mL, with approximately 163,000 live cells used per well. Microscopic images in Fig. 9 show the morphology of the seeded cell over 72 h. Cells initially appeared suspended, with adhesion observed by 24 h and increased attachment by 72 h in both scaffold types. The cells appeared in spheroid shape immediately after seeding, indicating they were suspended; by 24 h, the cells had started to adhere to the bone scaffold, and by 72 h, significant adhesion was observed in both scaffold types. Automated scaffolds consistently supported higher cell viability and robust adhesion.

## Discussion

Implementing the automated casting procedure using the Lulzbot bioprinter yielded significant improvements in scaffold fabrication compared to manual casting methods. The automated casting process was more consistent in scaffold weight and texture, while manually cast molds varied in these factors, with some molds appearing soggy and inconsistent in weight or color. This disparity indicates that automation is superior in scaffold manufacturing reproducibility.

Automated scaffold fabrication significantly improved efficiency and reproducibility. The automated casting with a 2.5 mL syringe was over 3.7 times faster than manual casting (*p* < 0.05), while casting with a 5 mL syringe was over 5.6 times faster (*p* < 0.01), showing statistically significant increases in throughput and manufacturing efficiency. The motivation behind substituting the casting process is to enhance the consistency and efficiency of the fabrication procedure, as reproducibility and scalability are essential in the biomedical field. The increased speed achieved through automation is advantageous for scalable production, critical in clinical and research applications where volume is needed [11, 40].

Automated casting saved time, and the coating was thicker and of higher quality. Scaffold weights from automated casting averaged 0.02354 g, whereas manual casting weights averaged 0.01169 g, a statistically significant difference (*p* < 0.01), indicating increased retention of the PLGA–HA coating with automated casting. These results showed a significant increase in scaffold retention, resulting in over twice the amount of PLGA–HA coating adhering to the PVA mold. Automated efficacy contributes to this marginal difference, as does the metallic mesh base, where the scaffolds were placed, removing the need for flipping for full coverage required in manual casting.

FTIR analyses confirmed the chemical integrity of the scaffolds. Characteristic peaks attributed to PLGA and HA were observed in both automated and manually cast samples, such as the 2953 cm^−1^ peak, indicative of C–H bonds in the methyl (–CH_3_) groups of PLGA, and the 1751 cm^−1^ peak for carbonyl (C=O) stretching vibration in PLGA’s ester groups. Additionally, HA’s presence was confirmed by the peak at 1455 cm^−1^, attributed to O–H bond bending vibrations in HA hydroxyl groups. The 1026 cm^−1^ and 875 cm^−1^ peaks represent phosphate (PO_4_^3−^) vibrations in HA. These results validate that automated casting retained the scaffold’s chemical integrity, ensuring consistency in biomedical applications without introducing any contaminants [41, 42].

SEM imaging further revealed differences in scaffold microstructure and morphology. The automated scaffolds exhibited consistency in pore size, with a range from 390 to 710 µm, whereas manually cast scaffolds showed a broader range of 251 µm to 1088 µm, as well as irregular strands and a non-uniform overall structure. Consistency in pore size and HA nanoparticle distribution are vital for stiffness and load-bearing capacities, as well as for better cell proliferation due to a more predictable surface texture. Inconsistencies found in manual casting may compromise mechanical integrity, reducing support and limiting cell adhesion and proliferation [41].

Compression tests revealed that the maximum stress before failure was 1.6 ± 0.08 MPa for both methods, with the automated scaffolds achieving an elastic modulus of approximately 0.77 MPa and manual scaffolds achieving 0.48 MPa. These results support the increased structural integrity of automated scaffolds. Both values agree with Hollister’s evaluation of the 0.4 MPa minimum elastic modulus when supporting cell culturing and other in vitro activities. These results support the increased structural integrity of automated scaffolds [5]. These findings are in alignment with SEM imaging, where uniform particle distribution in automated scaffolds contributes to fewer weak points, enhancing load-bearing capabilities.

Cell viability and adhesion were observed in both types of scaffolds. Cells seeded on both manual and automated scaffolds appeared in spheroid shape immediately after seeding, indicating suspension. By 24 h, cells started to adhere to the bone scaffold and flatten, and after 72 h, a significant number of cells exhibited robust adhesion and spreading throughout both sets of scaffolds. The consistent pore structure and surface topography of automated scaffolds contribute to a supportive environment for cell attachment and proliferation, maintaining high cell viability and robust adhesion [41].

The integration of automated casting with bioprinting technology demonstrated enhanced scaffold fabrication, improving consistency, efficiency, and reproducibility without compromising scaffold quality. Automated casting provided a fivefold time reduction and produced thicker, higher-quality coatings, which have potential applications in clinical and research environments, where consistency and scalability are essential. This approach enables reproducible fabrication of bioactive scaffolds, which is crucial for preclinical testing and scalable tissue engineering applications.
